# The role of surgical navigation in computer-assisted mandibular reconstruction: is it necessary?

**DOI:** 10.4317/medoral.26762

**Published:** 2024-10-13

**Authors:** Hui Yuh Soh, Shi Yue Shen, Yao Yu, Shuo Liu, Lei Hao Hu, Xin Peng, Wen Bo Zhang

**Affiliations:** 1 Department of Oral and Maxillofacial Surgery, Peking University School of Stomatology, Beijing, People’s Republic of China; 2 Department of Oral and Maxillofacial Surgery, Faculty of Dentistry, University Kebangsaan Malaysia, Kuala Lumpur, Malaysia; 3 Department of Oral and Maxillofacial Surgery, Peking University Shenzhen Hospital, Guangdong, People’s Republic of China

## Abstract

**Background:**

Accurate mandibular reconstruction following tumor ablation is important yet challenging. While computer-assisted surgery and surgical navigation have been applied widely in maxillofacial reconstruction, the accuracy and the efficacy remain debaTable due to the native mobile nature. This study aimed to evaluate the surgical outcomes and accuracy of mandibular reconstruction aided by different types of adjunctive computer-assisted techniques with or without intraoperative navigation.

**Material and Methods:**

Patients with anterior and/or lateral mandibular defects who underwent microvascular mandibular reconstruction aided by adjunct computer-assisted techniques, with or without intraoperative navigation were assessed. The deviations in spatial alignment of the bony segments between the pre-operative and post-operative datasets were measured to evaluate the overall surgical outcomes and accuracy. Independent t-test was performed and *p-value* less than 0.05 was statistically significant.

**Results:**

A total of 93 patients with L/LC or LCL defects who underwent microvascular mandibular reconstruction aided by adjunct computer-assisted techniques with or without surgical navigation were assessed. No significant difference was observed when comparing the mean differences between the preoperative and postoperative intercondylar and intergonial distance in both navigation-assisted and computer-aided design/computer-aided manufacturing (CAD/CAM) groups. There were also no significant differences noted among the different mandibular defects, osteosynthesis plates and types of free flap.

**Conclusions:**

Accurate mandibular reconstruction following tumor resection can be achieved by incorporating intraoperative navigation and adjunctive methods such as computer-assisted techniques and our innovative device, mandibular fixation device.

** Key words:**Mandibular reconstructions, surgical navigation, computer-assisted surgery, head and neck neoplasm.

## Introduction

Tumor ablative surgeries involving the mandible often result in significant functional and aesthetic deformities to the patients, which require meticulous planning and laborious microvascular reconstruction. Mandible provides pivotal framework to the lower third of the face and its integrity is fundamental to maintain the patency of the airway. Failure to restore the mandibular continuity may result in occlusal derangement and mandibular deviation, causing substantial impairment in both forms and functions. Therefore, optimal three-dimensional position of a reconstructed mandible is profoundly important in maintaining the upper airway patency and lower facial contour, while ensuring preservation of the deglutition, speech and masticatory functions. Although navigation-assisted surgery has been incorporated in the maxillofacial reconstruction to improve accuracy and final surgical outcomes in the past decades, the accuracy of navigation-assisted mandibular reconstruction can be compromised as the dataset synchronization is largely affected by the native movable nature of the mandible (1,2).

Earlier study has identified the predictors of clinical outcomes affecting the accuracy of navigation-assisted maxillofacial reconstruction, and has also concluded that the three-dimensional (3D) position of mandibular condyle and angle were affected by the 3D position changes in the bony flap and types of osteosynthesis plates in mandibular reconstruction guided by intraoperative navigation alone (2). While intraoperative navigation system was shown to be effective in improving the accuracy of the mandibular reconstruction, the application of computer-aided techniques alone such as 3D printed models and pre-bending of the titanium plate was observed to have satisfactory surgical outcomes (3). Hence, whether intraoperative navigation system has any added values in mandibular reconstruction remains debaTable. The aim of this study is to assess the outcomes of the mandibular reconstruction aided by adjunctive techniques with or without intraoperative navigation system.

## Material and Methods

- Patient sample

This single-center, retrospective cohort study included patients who underwent microvascular free flaps of the mandibular region, under single surgical team at the Department of Oral and Maxillofacial Surgery, Peking University School and Hospital of Stomatology between January 2012 and December 2023. The inclusion criteria were as follows: 1. Patients aged more than 18 years old; 2. Primary or secondary reconstruction with vascularized free tissue transfer following condyle-preserving segmental mandibulectomy; 3. Integration of adjunctive methods such as computer-assisted design/computer-assisted manufacturing (CAD/CAM) for 3D models printing and pre-bending of the reconstruction plate; 4. Application of mandibular fixation device in reconstruction of the central and lateral mandibular defects; 5. Complete postoperative CT datasets. Incomplete postoperative CT datasets and flap loss were excluded.

In this study, the mandibular defects were classified using Jewer’s HCL classification of the mandibular defects. The central segment which involves the bilateral mandibular canines were classified as C, while the lateral defect that excludes the condyle was categorized as L. Hemimandibular defects that sacrificed the condyles were classified as type H defects, however, these were excluded in this study (4,5). Based on the types of mandibular defects and types of surgery, the patients were categorized into two groups: group A: reconstruction performed aided by CAD/CAM and surgical navigation; and group B: reconstruction performed assisted by CAD/CAM only.

Free flap selection was ascertained based on the defect size, the general condition and relevant past medical history. While free fibula flap is a versatile workhorse flap in mandibular reconstruction, the use may be limited in patients with peripheral vascular diseases, particularly in elderly. Although DCIA flap is known to provide exceptional bone volume to accommodate osseointegrated implants, the use of DCIA flap may be avoided in obese patients due to the bulkiness of the myocutaneous components. Patients’ data including age, gender, diagnosis, surgical procedure, microvascular complications, flap success rate, and the use of surgical navigation were recorded into a Microsoft Office Excel 2019 (Microsoft, USA) spreadsheet.

- Image data acquisition and preoperative virtual surgical planning

In this study, all patients underwent preoperative head and neck contrast-enhanced computed tomography (CT) scan, as well as spiral CT scan for the lower extremities, either fibula or pelvis (ilium). (Field of view, 20cm; pitch 1.0; slice, 0.75mm; 120KV, 280mA).

**Figure 1:**
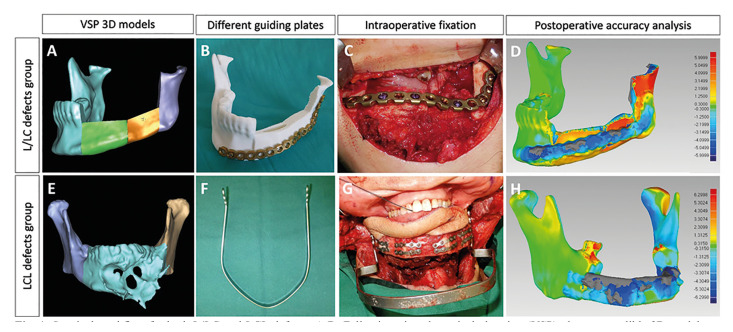
Surgical workflow for both L/LC and LCL defects. A-D: Following virtual surgical planning (VSP), the neomandible 3D model was printed into patient-specific neomandible model. Reconstruction plate was manually adapted on the model prior to sterilization. The pre-contoured reconstruction plate served as a guiding plate in the positioning of the osseous flap intraoperatively. Congruity analysis was performed based on the selected area of interest on both preoperative and postoperative iliac segments, showed in color map. E-H: In LCL defects group, the mandibular fixation device (Cibei, China) used in this study is a horseshoe shaped malleable titanium plate, which can be molded according to different patient’s mandibular width to allow passive adaptation of the 2 arms on ascending ramus. The mandible was positioned in centric relation and fixation of mandibular fixation device was performed prior to mandibulectomy. Color map analysis showed deviation between preoperative VSP and postoperative actual results.

In both groups, high resolution CT data were acquired in Digital Imaging and Communications in Medicine (DICOM) format and uploaded into the third party virtual surgical planning software. Following data conditioning and segmentation, volumetric models were created, and virtual surgical planning (VSP) were performed by the senior residents of the department, who had completed training course in virtual surgical planning, and had at least 3 years of clinical experience in head and neck reconstruction. The virtual surgical plan was subsequently verified by the chief surgeon, who had at least 5 years of experience in virtual surgical planning (Fig. 1). The mandibular reconstruction was guided by the original curvature of the mandible. However, in cases with apparent disruption of the original contour of the mandible, the reconstructions were performed via mirroring technique to reestablish symmetrical contour. The length, height and angle of each osseous flap segment were measured in the VSP software to facilitate flap harvesting and shaping intraoperatively. In group A, the final surgical plan was uploaded to navigation workstation, Kick® (BrainLAB, Feldkirchen, Germany) to facilitate tumor resection and to verify the spatial alignment of the bony segments intraoperatively.

In cases with the L/LC defects, following virtual surgical planning, the models of the reconstructed mandible were exported in Standard Tessellation Language (.stl) format for printing of 3D models, and UniLOCK Reconstruction Plate 2.4 (AOCMF; Synthes, Switzerland) was directly adapted on the models preoperatively prior to sterilization (Fig. 1).

- Surgical procedures

In both groups, a two-team approach was implemented, whereby the tumor resection, neck dissection or recipient vessels preparation, and the free flap harvesting were performed simultaneously. The operative techniques of mandibulectomy and reconstruction were similar in both groups.

In group A (CAD/CAM with surgical navigation), skin surface registration with the navigation system was performed using laser surface scanning (z-touch®), following mounting of dynamic reference frame using a self-tapping screw through a scalp incision. Each osteotomy plane was marked and validated using intraoperative navigation system. Following flap insetting, the position and orientation of each bony segment were verified using intraoperative navigation system prior to fixation. While in group B (CAD/CAM group), the flap insetting completed based on surgeon’s clinical experiences.

The mandibular fixation device (Cibei, China) used in this study for fixation of the LCL defects is essentially a horseshoe shaped titanium plate with 2 arms on each end, providing 3 screw holes for fixation on the ascending ramus of the mandible (Fig. 1). The malleable titanium plate can be adjusted according to different patient’s mandibular width and curvature of the ascending ramus to allow passive adaptation of the 2 arms on ascending ramus. The mandible was positioned in maximal intercuspation and fixation of mandibular fixation device was performed prior to mandibulectomy. To prevent overclosure post-mandibulectomy, a caliper was used to measure the distance between the mandibular fixation device and sTable soft tissue landmarks such as nasal tip.

The surgical margins were determined based on the diagnosis, clinical and radiological presentation of each patient. In general, the surgical margins for cases with benign tumors were 1.0cm to 1.5cm, while the safety margins were extended to 2.0cm in malignant tumors. In cases with marked involvement of the inferior alveolar nerve, the resection margins were ensured to include the entire course of the inferior alveolar nerve. However, in cases diagnosed with osteosarcoma of the mandible, the safety margins were further extended to 2.5cm to 3.0cm. Frozen section pathology was conducted in cases with soft tissues involvement.

- Postoperative congruity analysis and follow-up

**Figure 2:**
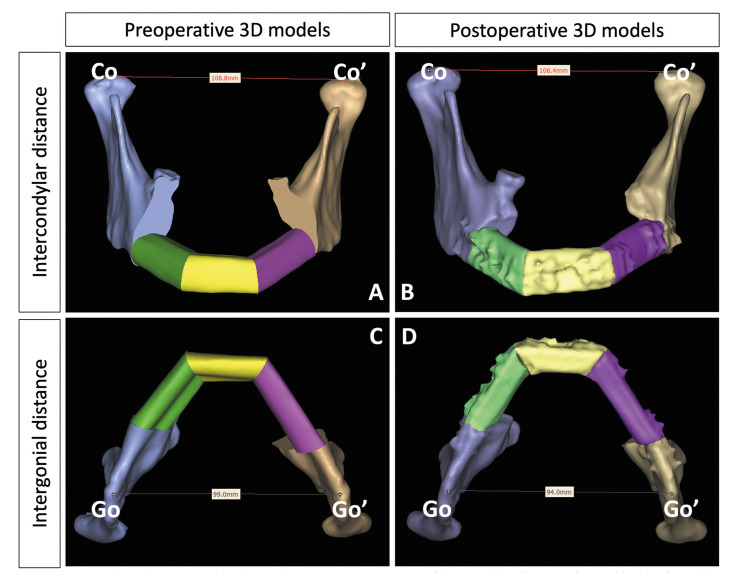
Preoperative and postoperative data analyses. A-B: Measurement of intercondylar distances from midpoint of condylar head to contralateral condylar head. The contralateral side was marked with apostrophe. C-D: Intergonial distance was measured from midpoint of gonion to another on the 3D model caudally. The contralateral side was marked with apostrophe.

Postoperative facial CT scan was obtained at 7 to 10 days after the surgery to assess the surgical outcomes and accuracy. The datasets were compared with preoperative virtual plan via superimposition and chromatography analysis using Geomagic Studio 2012 (3D System, USA). The mean, standard deviation, and root mean square (RMS) estimates of surface deviations between both pre-operative and post-operative models were generated automatically by the software. The intergonial and intercondylar distances were also measured using 3D distance measurement tool on PROPLAN CMF® 3.0 (DePuy Synthes, Solothurn, Switzerland and Materialise, Leuven, Belgium). Intergonial distance was measured from midpoint of gonion to another on the 3D model caudally. Intercondylar distance was measured from midpoint of condylar head to contralateral condylar head. The contralateral side was marked with apostrophe (Fig. 2).

All parameters were measured two raters (Soh H.Y. and Liu S.), taking the average of three measurements. The intra-rater and inter-rater reliability were evaluated using the intra-class correlation coefficients (ICC), and good reliability was achieved in all measurements (inter-rater, ICC=0.94-0.96, intra-rater, ICC=0.95-0.96).

Any post-operative adverse events or complications, including anastomotic related complications included venous thrombosis or arterial spasm, wound dehiscence, surgical site infection, flap failure, malocclusion, trismus and temporomandibular joint dysfunction were documented. The surgical complications were categorized according to the Clavien-Dindo classification (6).

- Statistical analysis

Statistical analyses were conducted using SPSS 24.0 (IBM, Armonk, NY). Continuous variables such as the intergonial and intercondylar distances were expressed as means standard deviations and were compared by the independent samples t-test. P-value less than 0.05 was considered statistically significant.

## Results

- Patient demographics

A total of 93 patients who fulfilled the inclusion criteria were included in this study, amongst all, 47 cases were L/LC defects, while 46 cases were LCL defects. Out of 93 cases, 48 cases were performed under the guidance of CAD/CAM and surgical navigation, while 45 cases were performed with the aid of CAD/CAM only. Among the patients enrolled in this study, 45 cases were of benign pathology, with 32 cases of ameloblastoma. Other benign pathologies included ossifying fibroma, myxoma, osteoradionecrosis of the jaw, and central giant cell granuloma. Squamous cell carcinoma constituted most of the malignant pathologies in this case cohort (33 cases), while other malignancies included osteosarcoma, mucoepidermoid carcinoma, and clear cell carcinoma. 52 patients underwent mandibular reconstruction with vascularized deep circumflex iliac artery flap (DCIA) and 41 patients received free fibula flap (FFF). The demographic data and surgical details were summarized in Table 1.

- Postoperative follow-up and complications

The mean patient age was 45.8±16.2years (range from 10 to 77 years of age) and the follow-up period was 23.6 months (range, 3 to 121 months). There were no statistically significant differences between the groups for gender or age. The overall flap success rate was 94.1% in this patient series. Two patients experienced anastomotic complications, in which one patient experienced venous congestion, immediate surgical re-exploration was performed, however, an attempt for re-anastomosis was abandoned as there was a marked thrombotic occlusion. The skin paddle was removed eventually, while the DCIA bone grafts remained. Another patient experienced arterial spasm and immediate surgical re-exploration and the flap survived after the arterial re-anastomosis. One patient suffered from soft tissue infection at post-operative one month which was resolved following surgical debridement and intravenous antibiotics treatment (Clavien-Dindo Grade IIIb). Severe malocclusion, trismus and temporomandibular joint dysfunction were not reported in this series of patients throughout the follow-up period. All patients were able to resume normal swallowing, speech, and masticatory functions as tolerating.

- Postoperative surgical outcomes and accuracy analysis

In general, group A (CAD/CAM with surgical navigation) demonstrated slighter difference when comparing pre-operative and post-operative intergonial and intercondylar distances as compared to the group B (exclusively CAD/CAM). The mean difference of pre- and post-operative intercondylar and intergonial distances in group A were 1.28±3.58mm and 0.11±4.87mm respectively; while in the group B, the intercondylar and intergonial distances measured were 2.46±6.72mm and 2.31±6.14 correspondingly. However, there was no significant difference observed between both groups as shown in Table 2. (*P*=0.294; *P*=0.062) The RMS estimates of group A and group B were 2.69±0.41 and 2.98±1.02 respectively, there was no significant difference noted between both groups. (*P*=0.109)

Comparison of the pre- and post-operative intergonial and intercondylar differences between different types of defects demonstrated greater intercondylar and intergonial differences pre- and post-operatively for L/LC defects, as compared to the LCL defects, however this did not reach significance. The mean difference of pre-operative and post-operative intercondylar and intergonial distances measured in L/LC and LCL defects group were 2.10±6.45 and 1.58±6.48, and 1.59±4.01 and 0.76±4.62 respectively (Table 3).

The mean difference of the intercondylar distance in reconstruction plate group was 1.42±6.18mm, while the mean intergonial difference was 0.73±5.85mm. In the miniplate group, the mean differences of the intercondylar and intergonial distance were 2.22±4.18mm and 1.50±4.93mm respectively (Table 3). However, there was no significant differences between miniplates and reconstruction plates in terms of the parameters measured above. There was also no significant difference noted in the mean differences of pre- and post-operative intercondylar and intergonial distances between types of free flaps, either the DCIA or free fibula flap (Table 3).

## Discussion

Advances in computer-assisted surgery and surgical navigation are instrumental in obtaining predicTable surgical outcomes and have revolutionized head and neck reconstruction at present. Most authors highlighted the accuracy of this technology in cases involving midface and skull base as these skeletons are fixed as a single unit. Bao *et al*. demonstrated better accuracy in mandibular reconstruction when combining CAS and surgical navigation. However, the degree of deviation was comparable to those performed without the aid of surgical navigation (7). Liu *et al*. introduced contour-registration based augmented reality system in the mandibular resection and reconstruction, which reduced the hand-eye incongruity and showed promising results. Nevertheless, the application was still limited to single segment fibular bone reconstruction, while multisegmented fibular reconstruction was more commonly used in routine clinical practice (8).

As the mandible is connected to the cranial base via temporomandibular joint, the indirect navigation tracking and the constant movements may compromise precise synchronization with the preoperative CT data intraoperatively. Thus, whether surgical navigation can further improve the computer-assisted mandibular reconstruction is yet debaTable. This study aimed to evaluate the necessity of surgical navigation in mandibular reconstructions aided by adjunctive methods. The current results showed that the adjunctive methods such as pre-bent plates and mandibular fixation device are able to improve the accuracy of mandibular reconstruction even without incorporation of the surgical navigation.

In general, maxillo-mandibular fixation (MMF) is the most commonly used method to enable temporary fixation of the mandible during the image-to-patient registration for the surgical navigation. The prerequisite for sTable MMF is to have sufficient remaining teeth on unaffected side, while in partially edentulous cases or in cases with extensive tumor involvement, the remaining teeth may be inadequate. As MMF may potentially block the surgical access, some authors prefer less invasive method, which is to position the mandible in a reproducible position manually or facilitated by occlusal splints. Casap *et al*. compared two different navigation systems for ablative surgery in mandible, both aided by occlusal template, and reported the navigational error of Image Guided Implantology (IGI) system (DenX Advanced Dental Systems, Moshav Ora, Israel) was less than 0.5mm, as compared to LandmarX system, and further concluded that teeth-retained sensor frame enables direct tracking of the mandible, which improves the accuracy of navigation in mandibular surgeries (9). While this maneuver is noninvasive, it can relatively tricky especially in edentulous patients or in patients with deranged occlusion secondary to extensive tumor involvement. The past clinical experience also showed that occlusal splint lacked adequate stability and rigidity in controlling the intergonial width, particularly in larger defects.

In this study, the pre-contoured reconstruction plate and mandibular fixation device served as a guiding template in insetting and positioning the bony flaps, while providing firm control of the transverse width of the mandibular segments and the condylar positions following segmental mandibulectomy. In cases that involved unilateral or central mandibular defects, the use of pre-contoured reconstruction plate is preferred; whereas in cases with angle-to-angle mandibular defects, the mandibular fixation device is favored. While intermaxillary fixation used to be the fundamental measure to maintain sTable occlusion in the past, this is no longer necessary as the pre-bent reconstruction plate is sufficiently rigid to maintain the width of the mandible, therefore allowing the surgeons to maintain the occlusion passively, and this could potentially overcome the issues mentioned prior.

Abbate *et al*. proposed direct mounting of dynamic reference frame on the mandible to allow maximum mandibular movements and reported the average standard deviation of 4.7mm (1). Although this method allows optical tracking of the mandible movements intraoperatively, it might not be feasible in larger defects or cases involving bilateral mandibular bodies, for instance, the central and lateral (LCL) defects. In this study, the use of mandibular fixation device utilized the remaining bony structures of the ascending ramus of the mandible to maintain the transverse width of the mandible, while allowing adequate mandibular movements intraoperatively.

Luhr technique which was first described in orthognathic surgical case, was applied in one of the mandibular reconstruction cases reported by Marchetti *et al*. This technique involved fixation of Luhr’s titanium positioning plate at maxillary tuberosity and coronoid of mandible to maintain centric condyle-fossa relationship (10). Nevertheless, this could be only feasible in unilateral mandibular defects with sufficient dentition.

Previous study had summarized various guiding plates for different mandibular defects and had shown promising results in using pre-bent reconstruction plate in reconstruction of unilateral mandibular defects using vascularized iliac crest flap (11,12). Several studies have also concluded that the accuracy of mandibular reconstruction was largely affected by the degree of fit of the patient-specific pre-bent titanium plate (13,14). In our opinion, the pre-bending of the reconstruction plate allows tension-free and passive adaptation to the mandible and improve the general fitting accuracy, while reducing total operating time and potential hazard of metal fatigue. Furthermore, in cases of advanced tumor extension with involvement of the buccal cortex or marked destruction of the original mandibular contour, intraoperative bending may not be feasible. Additionally, the presence of soft tissues and restricted access may further impede the passive adaptation of the plate, and the complex spatial conFiguration of the mandible may be overlooked intra-operatively.

Although the intraoperative navigation provides real-time visualization of the orientation of the flap to surgeons, the pre-bent reconstruction plate in fact serves as a guiding template, allowing translation of virtual surgical plan into actual reconstruction surgery, even without the utilization of the surgical navigation.

The average intergonial distances in our study were comparable with the study conducted by Naros *et al*., which compared the accuracy of mandibular reconstruction between pre-bent reconstruction plates and intraoperatively bent plates. A larger deviation in the intergonial distances was reported in the intraoperatively bent plates group as this could be attributed by the lack of anatomical reference during the reconstruction (14). In our study, the intergonial distances were generally slighter than the intercondylar distances, this could be contributed by the use of pre-bent reconstruction plate or the mandibular fixation device, which provides firm control of the transverse width of the mandible during reconstruction.

Maintaining transverse dimension is important in restoring original forms and functions, and this is particularly affected in LCL type of mandibular defects due to the muscular forces and loss of dentition. Failure to restore the transverse dimension may lead to malocclusion, TMJ dysfunction in long term. In such defects, the remaining dentition may not be sufficient to provide sTable occlusion and especially in partially dentate or edentulous patients, fabrication of occlusal splint or archbars placement may not be feasible. Marchetti *et al*. illustrated double-plate pre-plating technique to maintain the original position of the mandible (10), in which the first plate acts comparably to the mandibular fixation device as described previously. Likewise, Susarla *et al*. used Synthes MatrixMANDIBLE 2.0mm titanium straight plate to maintain intergonial width by plating directly at the inferior border of mandible (15). Shen *et al*. described a novel method in reconstructing angle to angle mandibular defect using mandibular fixation device and achieved excellent results with largest deviation of 2.441mm (16). In the present study, we incorporated the mandibular fixation device and observed that it had allowed firm control of the remaining proximal segments, the degree of deviation of intergonial and intercondylar distance was limited, thus giving a better morphological outcome as compared to the L/LC group.

Kim *et al*. reported increased error in condylar position in FFF which might be due to the thinner dimension of fibula as compared to DCIA, and concluded the superiority of DCIA over FFF in reproducibility of surgical guide and postoperative stability after 6 months post-operatively (17). The current results showed no significant difference between types of free flaps in terms of postoperative outcomes conversely. Although the present study mainly focused on evaluation of post-operative outcomes and accuracy, there was no reported condylar displacement in all the patients throughout the follow-up period.

The authors acknowledged some inherent limitations within the present study. This is a retrospective study with a relatively small and non-homogenous cohort. The discrepancies during data fusion and substandard image data resolution may have further contributed to the inaccuracies. In this study, the types of mandibular defects were limited to central and lateral mandibular defects that exclude the condyle, as the current adjunctive methods were unable to control the condylar segment, while the methods to control the condylar segment is beyond the scope of the discussion within this context.

## Conclusions

Accurate reconstruction of mandibular defects following tumor ablation can be challenging. The present study demonstrated that firm control of the remaining proximal segments is essential in improving the accuracy of the mandibular reconstruction. While intraoperative navigation system was shown to be effective in maxillofacial reconstruction in the past decade, this study shows optimal mandibular reconstruction can be achieved in condyle-preserving mandibular defects, even without the application of surgical navigation. However, it is essential to obtain high-resolution CT data to produce an accurate 3D model for the pre-bending of the reconstruction plate for sufficient aesthetic and functional reconstruction. The mandibular fixation device is particularly valuable in maintaining the transverse width of the mandible, thereby improving the overall surgical accuracy and post-operative surgical outcomes.

## Figures and Tables

**Table 1:** Demographic details.

Demographic details		Group A (n=48)	Group B (n=45)
Gender	Male	25	34
	Female	23	11
Age	≤ 50 years old	22	23
	> 50 years old	26	22
Site	L/LC	29	18
	LCL	19	27
Diagnosis	Benign	32	12
	Malignant	16	33
Types of reconstruction	FFF?	15	26
	DCIA‡	33	19
Types of osteosynthesis plate	Miniplates	15	27
	Reconstruction plate 2.4	33	18

Group A incorporated adjunctive techniques along with CAD/CAM and surgical navigation; Group B utilized adjunctive techniques with CAD/CAM only; ? Free fibular flap; ‡ Deep circumflex iliac artery flap.

**Table 2:** Comparison of the deviation of condylar and gonial positions in both groups using independent t-test.

Comparison		Group A	Group B	P-value
Intercondylar distances	Pre-op	110.40±6.61	108.33±6.59	0.294
	Post-op	109.12±6.46	105.86±9.72	
	Differences	1.28±3.58	2.46±6.72	
Intergonial distances	Pre-op	98.47±7.39	97.43±8.41	0.062
	Post-op	98.37±8.56	95.12±6.97	
	Differences	0.11±4.87	2.31±6.14	
RMSE ± SD		2.69±0.41	2.98±1.02	0.109

Group A incorporated adjunctive techniques along with CAD/CAM and surgical navigation; Group B utilized adjunctive techniques with CAD/CAM only; *P-value < 0.05 was considered statistically significant.

**Table 3:** Comparison of the condylar and gonial deviations in between different mandibular defects, types of free flaps, and the types of osteosynthesis plates.

Comparison		Intercondylar distances				Intergonial distances			
		Pre-op	Post-op	Differences	P-value	Pre-op	Post-op	Differences	P-value
Types of defects	L/LC defect	108.63±6.65	106.53±9.91	2.10±6.45	0.656	98.49±7.69	96.92±7.66	1.58±6.48	0.487
	LCL defect	110.15±6.64	108.56±6.33	1.59±4.01		97.47±8.09	96.72±8.33	0.76±4.62	
Types of free flaps	DCIA	108.31±6.60	106.55±9.63	1.76±6.23	0.863	98.19±7.87	96.91±7.69	1.29±6.32	0.816
	FFF	110.73±6.54	108.78±6.26	1.95±4.04		97.71±7.96	96.69±8.37	1.01±4.65	
Types of osteosynthesis plate	Reconstruction plate	108.19±6.54	106.78±9.58	1.42±6.18	0.488	97.66±7.69	96.93±7.60	0.73±5.85	0.515
	Miniplate	110.52±6.72	108.30±6.97	2.22±4.18		97.86±7.92	96.36±8.61	1.50±4.93	

? Free fibular flap; ‡ Deep circumflex iliac artery flap; *P-value < 0.05 was considered statistically significant.

## References

[1] Ghai S (2022). Ameloblastoma: An updated narrative review of an enigmatic tumor. Cureus.

[2] Kreppel M, Zöller J (2018). Ameloblastoma-Clinical, radiological, and therapeutic findings. Oral Dis.

[3] Vered M, Wright JM (2022). Update from the 5th edition of the world health organization classification of head and neck tumors: Odontogenic and maxillofacial bone tumours. Head Neck Pathol.

[4] Evangelou Z, Zarachi A, Dumollard JM, Peoc'h M, Komnos I, Kastanioudakis I (2020). Maxillary ameloblastoma: A review with clinical, histological and prognostic data of a rare tumor. In Vivo.

[5] McClary AC, West RB, McClary AC, Pollack JR, Fischbein NJ, Holsinger CF (2016). Ameloblastoma: a clinical review and trends in management. Eur Arch Otorhinolaryngol.

[6] Minichetti JC, D'Amore JC, Schwarz E (2011). Complete oral rehabilitation of a postresection ameloblastoma patient: a clinical case report. J Oral Implantol.

[7] Donkor P, Bankas DO, Boakye G, Ansah S, Acheampong A (2006). The use of free autogenous rib grafts in maxillofacial reconstruction. Ghana Med J.

[8] Bibbo C, Nelson J, Ehrlich D, Rougeux B (2015). Bone morphogenetic proteins: indications and uses. Clin Podiatr Med Surg.

[9] Zekry KM, Yamamoto N, Hayashi K, Takeuchi A, Alkhooly AZA, Abd-Elfattah AS (2019). Reconstruction of intercalary bone defect after resection of malignant bone tumor. J Orthop Surg.

[10] Xu L, Li C, Wang H, Zhu S, Li Y (2019). Distraction osteogenesis of fibula graft for mandibular reconstruction following ameloblastoma ablation. J Craniofac Surg.

[11] Herford AS, Stoffella E, Tandon R (2011). Reconstruction of mandibular defects using bone morphogenic protein: can growth factors replace the need for autologous bone grafts?. A systematic review of the literature. Plast Surg Int.

[12] Page MJ, McKenzie JE, Bossuyt PM, Boutron I, Hoffmann TC, Mulrow CD (2021). The PRISMA 2020 statement: an updated guideline for reporting systematic reviews. BMJ.

[13] Munn Z, Barker T, Moola S, Tufanaru C, Stern C, McArthur A (2020). Methodological quality of case series studies: an introduction to the JBI critical appraisal tool. JBI Evid Synth.

[14] Simon EN, Merkx MA, Shubi FM, Kalyanyama BM, Stoelinga PJ (2006). Reconstruction of the mandible after ablative surgery for the treatment of aggressive, benign odontogenic tumours in Tanzania: a preliminary study. Int J Oral Maxillofac Surg.

[15] Li Z, Zhao Y, Yao S, Zhao J, Yu S, Zhang W (2007). Immediate reconstruction of mandibular defects: a retrospective report of 242 cases. J Oral Maxillofac Surg.

[16] Schlieve T, Hull W, Miloro M, Kolokythas A (2015). Is immediate reconstruction of the mandible with nonvascularized bone graft following resection of benign pathology a viable treatment option?. J Oral Maxillofac Surg.

[17] Marschall JS, Kushner GM, Flint RL, Jones LC, Alpert B (2020). Immediate reconstruction of segmental mandibular defects with nonvascular bone grafts: A 30-year perspective. J Oral Maxillofac Surg.

[18] Melville JC, Tran HQ, Bhatti AK, Manon V, Young S, Wong ME (2020). Is reconstruction of large mandibular defects using bioengineering materials effective?. J Oral Maxillofac Surg.

[19] Dastgir R, Coffey J, Quereshy H, Baur DA, Quereshy FA (2024). Nonvascularized bone grafts: how successful are they in reconstruction of segmental mandibular defects?. Oral Surg Oral Med Oral Pathol Oral Radiol.

[20] Clokie CM, Sándor GK (2008). Reconstruction of 10 major mandibular defects using bioimplants containing BMP-7. J Can Dent Assoc.

[21] Marechek A, AlShare A, Pack S, Demko C, Quereshy FA, Baur D (2019). Nonvascularized bone grafts for reconstruction of segmental mandibular defects: Is length of graft a factor of success?. J Oral Maxillofac Surg.

